# Vaccination of Sheep with Bovine Viral Diarrhea Vaccines Does Not Protect against Fetal Infection after Challenge of Pregnant Ewes with Border Disease Virus

**DOI:** 10.3390/vaccines9080805

**Published:** 2021-07-21

**Authors:** Gilles Meyer, Mickael Combes, Angelique Teillaud, Celine Pouget, Marie-Anne Bethune, Herve Cassard

**Affiliations:** 1 Interactions Hôtes-Agents Pathogènes (IHAP), Université de Toulouse, INRAE, ENVT, 31100 Toulouse, France; angelique.teillaud@envt.fr (A.T.); herve.cassard@envt.fr (H.C.); 2 Groupement Vétérinaire Saint Léonard, 87400 Saint Léonard de Noblat, France; mika-combes@hotmail.fr; 3 Fédération des Organismes de Défense Sanitaire de l’Aveyron, 12000 Rodez, France; celine.pouget.gds12@reseaugds.com; 4 RT1 Port Laguerre, BP 106, Païta, 98890 Nouvelle Calédonie, France; marieannebethune@gmail.com

**Keywords:** Border Disease Virus, sheep, vaccination, fetus, protection, Bovine Viral Diarrhea Virus

## Abstract

Border Disease (BD) is a major sheep disease characterized by immunosuppression, congenital disorders, abortion, and birth of lambs persistently infected (PI) by Border Disease Virus (BDV). Control measures are based on the elimination of PI lambs, biosecurity, and frequent vaccination which aims to prevent fetal infection and birth of PI. As there are no vaccines against BDV, farmers use vaccines directed against the related Bovine Viral Diarrhea Virus (BVDV). To date, there is no published evidence of cross-effectiveness of BVDV vaccination against BDV infection in sheep. We tested three commonly used BVDV vaccines, at half the dose used in cattle, for their efficacy of protection against a BDV challenge of ewes at 52 days of gestation. Vaccination limits the duration of virus-induced leukopenia after challenge, suggesting partial protection in transient infection. Despite the presence of BDV neutralizing antibodies in vaccinated ewes on the day of the challenge, fetuses of vaccinated and unvaccinated sheep were, two months after, highly positive for BDV RNA loads and seronegative for antibodies. Therefore, BVDV vaccination at half dose was not sufficient to prevent ovine fetal infection by BDV in a severe challenge model and can only be reconsidered as a complementary mean in BD control.

## 1. Introduction

The *Pestivirus* genus within the *Flaviviridae* family comprises eleven recognized species, named from A to K [1]. Along with Classical swine fever virus (*Pestivirus* C) and Bovine Viral Diarrhea Virus 1 and 2 (BVDV-1 or *Pestivirus* A, BVDV-2 or *Pestivirus* B), Border Disease Virus (BDV, *Pestivirus* D) belongs to one of the 4 main viral species recognized within this genus. BDV is responsible for Border Disease (BD), which is primarily an infection of sheep and rarely of goats and cattle, characterized by immunosuppression and increased risk of other infections, congenital disorders, abortion, stillbirths, and the birth of weak lambs persistently infected (PI animals) experiencing tremors, abnormal body conformation, and hairy fleece [2]. The occurrence of BDV infection in domestic and wild animals, mostly in sheep, has been confirmed in different countries worldwide, but most of the data come from Europe [3]. In France, the first case of BDV infection in sheep was reported in 1983 in the Aveyron department and was characterized by disease and high mortality in adults and lambs [4,5]. Since then, screening and prevention measures have been implemented at least in this region which has led to a gradual decline in the BD prevalence from 20% in 1998 to 4% in 2005. In 2009–2013, a resumption of BDV circulation was observed with severe clinical consequences in lambs [6]. Serological screening showed that in 2010 the average seroprevalence was 9.3%, with a significant difference between dairy (6% seropositive) and suckler (22% seropositive) herds. Again, control measures have been put in place by the breeders’ associations to limit BD spread [6]. These measures are traditionally based on the detection and elimination of PI lambs, biosecurity measures, and vaccination. In sheep farming regions, vaccination is currently used alone, mainly because there is no substantial financial support for PI animals’ detection and elimination. The objectives are to prevent clinical forms after transient infections and to prevent the birth of PI lambs by protecting the fetus from transplacental infection.

Currently, the only commercialized vaccines used in the field are BVDV ones, which do not contain BDV valences. For economic reasons they are used with half or sometimes a quarter of the dose used in cattle.

Several studies have assessed the cross serological response of sheep between BVDV and BDV after natural infection [7], challenge [8,9], or immunization with BVDV vaccines [10,11]. Cross serological neutralizing reactions were previously demonstrated between the BVDV-1 NADL strain and the BDV Moredun cytopathic one although the virus-neutralizing antibody titers (VNT) were lower than those obtained in the homologous vaccine test [7]. On the other hand, BVDV-1 and BVDV-2 were shown to be poorly or not neutralized by antisera generated after infection of sheep with BDV-1 strain V2536, BDV-2 strain17385, and BDV-3 strain Gifhorn [9]. Interestingly, the antisera raised against BDV-1, BDV-2, and BDV-3 strains neutralized heterologous BDV strains to the same extent. Finally, immunization of sheep with 7 BVDV vaccines induced the production of antibodies capable of neutralizing the BDV-1 Singer strain in 5 of them [10]. Similar results were obtained after the immunization of cattle with the same vaccines, although the VNT was lower than in sheep. In 2012, Anne [11] showed that ewes vaccinated at a half dose with a killed or a modified live virus (MLV) BVDV vaccine produced antibodies capable of neutralizing the BVDV-1 NADL strain and two BDV field strains of genotypes 3 and 5. However, there was significant individual variability in each test group and the heterologous neutralizing response (against BDV strains) decreased rapidly as early as three months post-vaccination while the homologous response (against BVDV-1) remained stable over the six month study period [11].

To date, there are no experimental data demonstrating the efficacy of BVDV vaccines to protect against BDV infections in a challenge model. Here, we tested the efficacy of the three commercialized BVDV vaccines, used for BD control, for fetal protection against a challenge with a non-cytopathic BDV genotype 6 strain recently isolated from a PI lamb during a BD epidemic in 2010 in central France.

## 2. Materials and Methods

### 2.1. Virus and Inoculum

The BDV non-cytopathic (ncp) strain 6390 was isolated in 2010 in France, Aveyron, from a PI lamb suffering from hairy shaker syndrome. It was previously shown to belong to BDV genotype 6 and to successfully produce 100% PI lambs when inoculated to naïve ewes at 52 days of gestation [12]. BDV-6390 inoculum was produced by five passages in bovine Madin Darby Kidney cells (MDBK, ATCC CCL-22). The titer of the inoculum was adjusted to 4 × 10^4^ TCID_50_/mL before the challenge.

### 2.2. Vaccination of Ewes and Artificial Insemination

**Figure 1:**
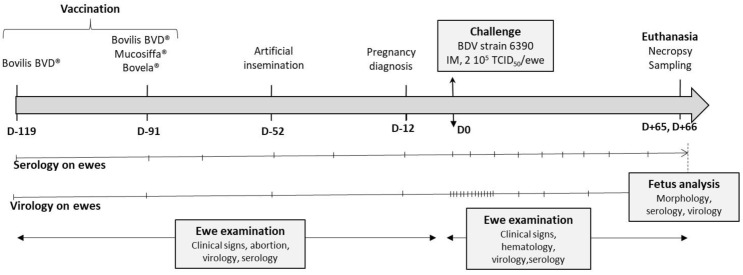
Experiment timeline of vaccination, artificial insemination, and BDV challenge and sampling.

Sixty Lacaune ewes were used, from two Aveyron BDV-free farms, which were all seronegative before vaccination by seroneutralization and NS2-3 ELISA (ID Screen BVD p80 Antibody One-Step^®^, ID Vet, Montpellier, France) and negative for pestivirus by RT-qPCR (pan-pestivirus RT-qPCR, IdVet, Montpellier, France) at the days of vaccination, of artificial insemination and of challenge. Twenty ewes were vaccinated by two injections of the Bovilis BVD^®^ inactivated vaccine (Bovilis, MSD Animal Health, Kenilworth, NJ, USA) at 119 and 91 days (D-119, D-91) before challenge (Figure 1), according to the manufacturer’s recommendations, except for the dose which was divided by half (1 mL, intramuscular route). On the day of the second Bovilis injection (D-91), two batches of 20 ewes, physically separated, were vaccinated with Mucosiffa^®^ (Mucosiffa, CEVA SA) or Bovela^®^ (Bovela, Boehringer Ingelheim, Ingelheim, Germany) vaccines in a single injection according to the manufacturer’s recommendations, except for the dose which was divided by half (1 mL, intramuscular route). In each farm, an additional batch of 20 unvaccinated ewes was used as controls and sentinels against wild BDV infection. These animals remained seronegative and BDV negative until the day of challenge.

At D-52 all ewes were inseminated. On the basis of an ultrasound pregnancy test, a total of 51 pregnant ewes were sent to the National Veterinary School of Toulouse (ENVT) at D-12 (Figure 1). Animals were housed in biosafety level 2 and the experiment was designed in conformance with the guidelines of the European Community Council on Animal Care (2010/63/EU) and under the authority of a license issued by the National Ethics Committee (APAFIS#6343-2016080817133467, French Ministry of Agriculture, Ethics Committee no. 115). At the ENVT, sheep were divided into three physically separated rooms, each containing 11 ewes vaccinated with one product as well as 6 non-vaccinated ewes. For further analysis, groups were defined as follows: group 1 with Mucosiffa vaccinated ewes (n = 11), group 2 with Bovela vaccinated ewes (n = 11), group 3 with Bovilis BVD vaccinated ewes (n = 11), and group 4 containing all non-vaccinated sheep (n = 18, from the 3 locations). A regular serological and virological follow-up was performed on all ewes before their arrival at the ENVT (Figure 1). The results showed an absence of NS3 antibody seroconversion in the control/sentinel ewes and an absence of BDV or BVDV detection by RT-qPCR in all ewes. At the ENVT, control animals remained seronegative until challenge. All ewes were negative for BDV or BVDV by RT-qPCR at D-12 and D0 of challenge.

### 2.3. Challenge and Sampling

All vaccinated and non-vaccinated ewes were challenged at D0 by intramuscular inoculation of 2 × 10^5^ TCID_50_/ewe (5 mL per ewe) of BDV-6390, at 52 days of gestation. The titer of the BDV inoculum was confirmed after the challenge. A complete clinical examination of the ewes was performed daily from D0 to D+20 following the experimental infection. Thereafter, daily monitoring (observation only) was performed until the end of the experiment, in particular, to detect possible abortion.

The hematological examination was performed on 6 ewes per vaccinated group and 9 non vaccinated ewes at D-3, D-2, D-1, D0 to D+4, D+6 to D+8, D+10, D+11, and D+13. Blood cell counts were performed on an MS9-5 analyzer (Melet Schloesing Laboratories) calibrated for sheep (ENVT biological analysis laboratory). Results were expressed as follows: for each animal and each cell population, a baseline was calculated as the average of the data obtained over the 3 measurements preceding viral inoculation (D-3, D-1, and D0). For each cell population, the individual data were transformed into the percentage of each cell population back to the baseline ((X/baseline)/100). Statistical analyses were performed on the log-transformed data.

An abdominal ultrasound was performed on each animal 1.5 months after challenge (D+45 to D+48) to assess fetal viability. All ewes were euthanized between D+64 and D+67 using an overdose of general anesthesia (5 mg/kg ketamine followed by 15 mg/kg pentobarbital sodium) and exsanguination. Fetuses and placenta were recovered, immediately after euthanasia, fetuses were measured (radius and tibia length, atlo-caudal distance) and examined for malformations. Fetal blood was collected by cardiac puncture for serology, then brain (cortex and cerebellum) and thymus were collected from each fetus for virological (storage at −80 °C) analyses.

### 2.4. Antibody Response

Pestivirus antibodies were detected in ewes at D-91, D-77, D-70, D-61, D-52, D-31, D-12, D0 and every 7 days until D+64 to D+67 and in all fetuses at euthanasia using a competitive ELISA (BVD p80 (NS2-3) antibody One-Step kit, IDvet, Montpellier, France). Serum neutralization assays (SN) of ewes were performed before vaccination (D-91), on D0 (day of challenge) and at the end of the experiment (D+66) on 5 ewes per vaccinated group and on 9 non vaccinated control ewes. The ability of sheep antibodies to neutralize BVDV was tested against the cytopathic strain (cp) BVDV-1a NADL and the ability to neutralize BDV against the ncp challenge strain BDV-6 6390 as already described [13]. For the ncp BDV-6 strain, infection was detected by immunoperoxydase assay (IPMA). Briefly, infected cells were fixed with paraformaldehyde 4% (20 min à 37 °C), permeabilized (PBS and Triton X-100, 2%; 60 min, 37 °C), saturated (PBS, skim milk 3%, 60 min, 37 °C) and then incubated with pan-pestivirus WB 103/105 (1/100 dilution, APPHA Scientific, Weybridge, UK) for 60 min at 37 °C. After washing, cells were incubated with a goat anti-mouse immunoglobulins (1/100 dilution, DAKO) for 60 min at 37 °C then revealed using an AEC detection method (AEC detection kit, Sigma, St. Louis, MI, USA). For the NADL strain, infected cells were detected on the basis of cytopathic effect and confirmed by IPMA. Virus neutralizing antibody titers (VNT) were expressed as the effective dose of 50% (ED_50_) calculated by the Spearman-Kärber method.

### 2.5. Virus Detection

BDV or BVDV detection was carried out on blood samples of ewes (D0 to D+15, D+21, D+28, D+35, D+42, D+49, D+56, and D+66) and on tissues samples of fetuses. RNA extraction from blood EDTA samples was performed directly after collection using the NucleoSpin RNA blood^®^ kit (Macherey-Nagel, Düren, Germany) according to the manufacturer’s recommendations. Tissue lysis (30 mg) was obtained by lysis in Precellys lysing kit tubes (P000912-LYSKO-A, Bertin Technologies, Montigny-le-Bretonneux, France) with 500 μL of Opti-MEM on a Precellys system (Bertin Technologies, France). RNA from tissues was extracted using the RNeasy kit (Qiagen, Hilden, Germany). BDV detection was performed by real-time quantitative ‘panpesti’ RT-PCR using the ID Gene™ BVD/BD Triplex kit (IDvet, Montepellier, France) in the LightCycler 96 Real-Time PCR System (Roche, Basel, Switzerland). Some positive tissues samples were isolated on MDBK cells to confirm infectivity and for further sequencing. The genomic region encoding the highly conserved 5′-NCR of the genome was amplified using the panpestivirus primers 324 and 326 flanking a 249-bases fragment [14]. Viral RNA was retro-transcribed using the RevertAid Reverse Transcriptase (Thermo-Fischer, Waltham, MA, USA) according to the manufacturer’s protocol. Then a 5-μL aliquot of cDNA was used as a template for PCR amplification with Taq DNA polymerase (QIAGEN, Hilden, Germany) kit, on Light Cycler 96 (Roche) using the profile of 94 °C for 3 min; 35 cycles (94 °C for 60 s; 55 °C for 60 s and 72 °C for 30 s); 72 °C for 10 min; and a 4 °C hold. Amplicons were separated using 2% agarose gel and were subsequently purified using QIAquick Gel Extraction Kit (QIAGEN, Hilden, Germany). Sequencing was performed by the Sanger method using the primers mentioned before (GATC Biotech services, Landkreis Ebersberg, France). Sequences were edited and compared with each other using the BioEdit software.

### 2.6. Statistical Analyses

Statistical analyses were performed using GraphPad (La Jolla, CA, USA). Logarithmic transformation was applied to fulfill the conditions of variances in homogeneity and normality when necessary (qPCR, hematology data). Data were expressed as arithmetic mean ± standard error of the mean (SEM) or standard deviations (SD). A two-way ANOVA with repeated measures (three-factor splitplot ANOVA) was used to analyze the qPCR results on blood samples of ewes, the VNT, and blood cell counts. When effects of the “day” and “treatment” factors were significant among interactions, a Bonferroni test between contrasts was used to compare the treatments on each day post-challenge. Levels of significance are indicated on the graphs with letters when *p* < 0.05. A one-way ANOVA was used to compare the BDV detection in fetuses. When the effect of the “treatment” factor was significant, a Newman-Keuls test was used to compare the treatment effects at each time point.

## 3. Results

### 3.1. The Two Attenuated BVDV Vaccines But Not the Inactivated One, Induced Seroconversion against NS3 in Sheep

**Figure 2:**
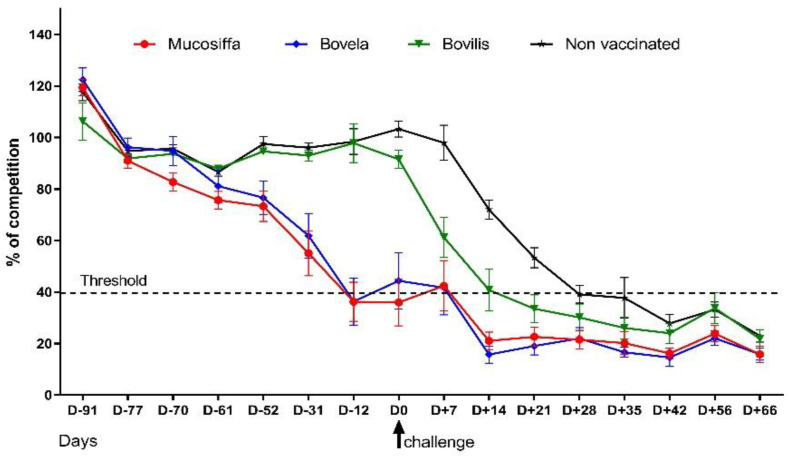
Mean ELISA antibody competitive percentages calculated according to the manufacturer’s recommendations (OD sample/mean OD of negative controls, validation of the assay if the mean OD of negative controls > 0.7 and the OD of the positive control is less than 30% of the mean OD of negative controls: OD positive control/mean OD of negative controls < 0.3). Interpretation: positive: ≤40%; doubtful: >40% and ≤50%; negative: >50%.

Before vaccination, all ewes were seronegative for NS3 antibodies. Non-vaccinated ewes remained seronegative until challenge (Figure 2). They seroconverted between D+21 (3/19 positives) and D+42 (17/19 positives) post-infection and all animals were seropositive at, at least, one sampling point. While sheep vaccinated with Bovilis were also negative at D0, they started seroconversion after challenge earlier: between D+7 (2/11 positives) and D+35 (10/11 positives). One ewe of this group remained seronegative during the entire experiment.

Vaccination with the two attenuated BVDV vaccines induced a seroconversion which was delayed compared to the experimental infection in control sheep (Figure 2, Table 1). Seroconversion in the Mucosiffa group started 39 days post-vaccination (D-52) for one animal and 9 ewes out of 11 were seropositive for NS3 antibodies at 91 days post-immunization (D0). Sheep vaccinated with Bovela seroconverted from 60 days (D-31, 3/11 positives) to 91 days (D0, 7/11 positives) post-immunization. The BDV-6 challenge induced an anamnestic response in vaccinated ewes which were seropositive at D0, with percentages of competition falling from 9.7% to 34.2% (threshold of positivity: <40%) between D+7 to D+14. For Bovela and Mucosiffa sheep which were seronegative at D0, all animals seroconverted at D+14 post-infection. While the competition percentages do not reflect an actual titration, they do provide an assessment of the level of antibody response. At D0 the average competition percentages were 21.2%, 18.9%, and 91.8% for the Mucosiffa, Bovela, and Bovilis groups, respectively. After challenge the mean percentages varied between 19.8% and 12.6%; 21.4% and 9.8%; 27.9% and 18.3%; 35.4% and 15.1% for Mucosiffa, Bovela, Bovilis and control groups, respectively.

Finally, it appears that a small number of ewes, vaccinated or not, appear seronegative for NS3 antibodies after the challenge. This was the case for one ewe in the Bovilis group which remained seronegative throughout the experiment and for 1, 1, 3, and 7 ewes in the Mucosiffa, Bovela, Bovilis, and control groups respectively, which appeared seronegative intermittently at different sampling times between D+35 and D+66.

### 3.2. The Three Vaccines Induce a Neutralizing Antibody Response against BVDV-1 and BDV-6

**Figure 3:**
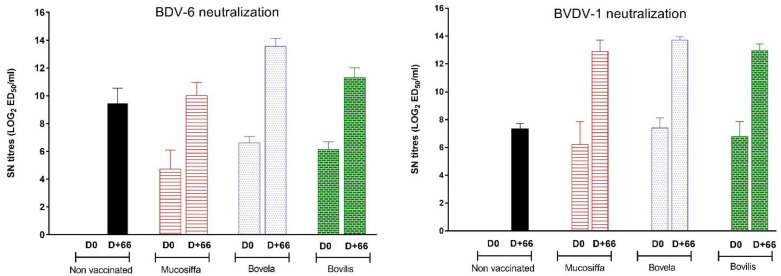
Mean neutralizing antibody titers (VNT) calculated by the Spearman-Karber method and expressed in log_2_. Sera from D0 and D+66 of vaccinated (Mucosiffa in red, Bovela in blue, Bovilis in green) and unvaccinated (in dark) ewes were tested against the BVDV-1a NADL and the BDV-6 6390 strains for neutralization. Standard errors of the mean are indicated.

As for the ELISA, the 9 non-vaccinated control ewes remained seronegative by SN assay until challenge. They subsequently seroconverted to have a mean VNT at D+66 of 9.4 ± 2.7 and 9.8 ± 1.6 Log_2_ ED50/mL against BDV and BVDV-1, respectively (Figure 3). While ewes vaccinated by Bovilis were negative for NS 2-3 ELISA antibodies at D0, they were positive for neutralizing antibodies, with VNT of 6.1 ± 1.2 and 6.8 ± 2.4 Log_2_ ED50/mL when tested against BDV and BVDV-1, respectively. In this group, the challenge induced an anamnestic response with VNT of 11.3 ± 1.6 and 12.9 ± 1.4 Log_2_ ED50/mL against BDV and BVDV-1 at D+66. Concerning sheep vaccinated with Mucosiffa, 4 ewes out of the 5 tested had neutralizing antibodies to BDV and BVDV-1 on D0, 91 days after vaccination. At the end of the experiment, all the ewes were positive in neutralizing antibodies, irrespective of the virus used. The mean VNT at D0 were 3.8 ± 3 and 5 ± 4.5 Log_2_ ED50/mL against BDV and BVDV-1, respectively while they increase after challenge with final titers at D+66 reaching 10 ± 2.1 and 12.5 ± 2.3 Log_2_ ED50/mL. The 5 ewes vaccinated with Bovela had neutralizing antibodies to BDV and BVDV-1 on D0, with a mean VNT of 6.6 ± 1 and 7.4 ± 1.6 Log_2_ ED50/mL against BDV and BVDV-1, respectively. The challenge also induced an increase in VNT at D+66, of 13.9 ± 1.6 and 13.7 ± 0.5 Log_2_ ED50/mL against BDV and BVDV-1, respectively.

Differences in VNT were significant (ANOVA) at D0 between non-vaccinated and infected ewes for both BVDV-1a NADL and BDV-6 SN assays. At D0, VNT were slightly higher when sera were tested against BVDV-1a than BDV-6, irrespective of the vaccine used, but differences were not statistically significant. Vaccination with Bovela induced significantly higher mean VNT than Mucosiffa at D0 only for the BDV-6 SN assay. At D+66 the only significant differences were observed when Bovela was compared to Mucosiffa or non-vaccinated groups for the BDV-6 SN assay.

### 3.3. Vaccination Partially Protects Ewes against BDV-6 Challenge-Induced Leukopenia

**Figure 4:**
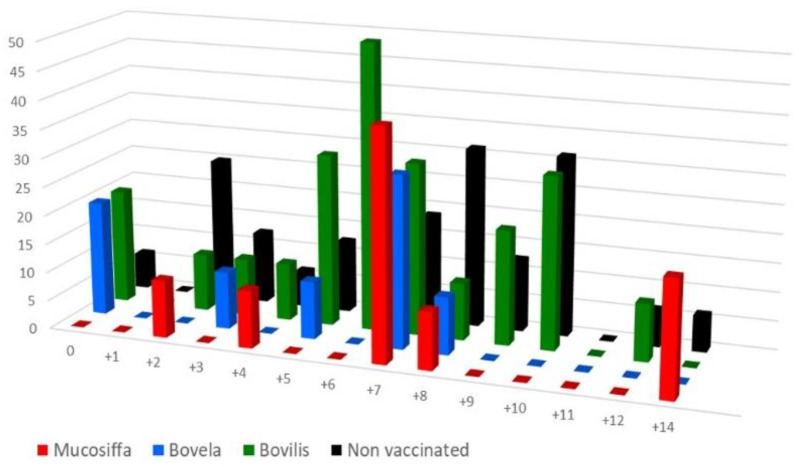
Percentage of BDV-6 positive animals, detected by real-time RT-qPCR in the blood of vaccinated (Mucosiffa in red, Bovela in blue, Bovilis in green) and unvaccinated (in dark) ewes after challenge.

Confirmation of the BDV-6 challenge was done by serological analyses and virus detection in the blood of inoculated ewes. The virus was detected, by two independent analyses, between D+3 and D+9 in the blood of 9, 8, 11, and 18 ewes of the Mucosiffa, Bovela, Bovilis, and control groups, respectively (Figure 4). Confirmation was carried out by short sequencing of 6 positive samples with Ct values between 21.5 and 25 (data not shown). The duration of detection was short, between 1 and 2 days per ewe, regardless of the group. There were no major differences between groups. Despite infection, we did not observe clinical signs in all ewes after the BDV-6 challenge. Similarly, no abortions were observed in the vaccinated ewes during the experiment. In the non-vaccinated group, one ewe aborted at D+55. The fetus was positive by RT-qPCR for BDV and negative by bacterial isolation for *Listeria monocytogenes* (Public Departmental Veterinary diagnostic LVD82, Montauban, France), by qPCR for *Coxiella burnetii*, *Chlamydophila abortus*, *Salmonella abortus* (Scanelis diagnostic laboratory, Colomiers, France), by qPCR for *Brucella abortus (*ID Gene^®^ Brucella spp triplex, IDvet, Montpellier, France), and by qPCR for *Toxoplasma gondii* (qPCR, VetMax T. gondii Kit, Thermo Fischer).

**Figure 5:**
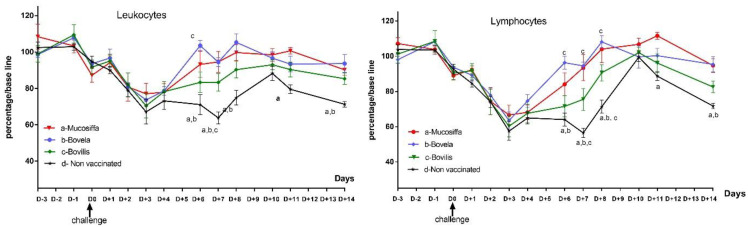
Mean leukocytes (white blood cell) and lymphocyte counts (with standard error of the mean) of the vaccinated (red line: Mucosiffa; blue line: Bovela; green line: Bovilis BVD) and non-vaccinated (black line) groups after BDV-6 challenge. For each animal and each cell population, a baseline was calculated as the average of the data obtained over the 3 measurements preceding viral inoculation (D-3, D-1, and D0). The individual data were then transformed into the percentage of each cell population back to the baseline ((X/baseline)/100). Significant differences in the mean number of leukocytes or lymphocytes between the group of the line and the other groups corresponding to a letter are indicated. Significant differences in the mean number of leukocytes or lymphocytes between the group corresponding to the line and the other groups designated by their respective letters are indicated.

Hematology results are presented for each group and day as the average of the percentage of each cell population brought to a baseline (Figure 5). Leukopenia was observed in all groups between D+1 and D+4, with average peak losses ranging from 23% (Mucosiffa group) to 33% (control group) of leukocytes at D+3. Leukopenia persisted until D+14 in the control group. In the Mucosiffa and Bovela groups, a rapid rise in leukocytes to baseline was observed between D+4 and D+6. The rise in leukocytes was more gradual for the Bovilis group, between D+6 and D+10. Statistically significant differences are shown in Figure 5. Leukopenia was mainly explained by lymphopenia. The decrease in mean lymphocyte percentage was most severe at D+3 for all BDV-6 infected groups, from −32% (Mucosiffa group) to −43% (control group) from baseline. Again, more rapid recovery of lymphocyte counts was observed in the Mucosiffa and Bovela groups. Significant differences in cells counts were mainly observed between the control and the two Mucosiffa and Bovela groups between D+6 and D+10 (Figure 5). No differences were observed between vaccinated groups except between Bovela and Bovilis groups at D+6 for leukocyte and D+6, D+7 and D+8 for lymphocyte counts. Regarding platelet counts, no differences were observed between the 4 groups (data not shown).

### 3.4. Vaccination with Half Dose of BVDV Vaccines Does Not Protect against Fetal Infection by BDV

The number of fetuses, between 1 and 4 fetuses per ewe, recovered at the end of the experiment reached 20, 22, and 21 for the Mucosiffa, Bovela, and Bovilis groups (11 ewes per group) respectively, and 41 for the control group (18 ewes). Only one malformed fetus was observed in the Bovela group, showing cerebral atrophy with poorly developed convolutions. The average fetal weights were 1.58 ± 0.23 kg, 1.64 ± 0.3 kg, 1.72 ± 0.23 kg and 1.60 ± 0.26 kg for the Mucosiffa, Bovela, Bovilis and the control groups, without significant differences. Similarly, the atlo-caudal distance (between 31.1 and 31.8 cm) and the lengths of the radius (between 6.5 and 6.7 cm), and the tibia (between 7.7 and 8.7 cm) were similar between the fetuses of the 4 groups without differences.

Testing for NS3 antibodies on fetal heart blood showed that all fetuses were seronegative except for a single fetus in the Bovela group that was tested seropositive with a competition percentage of 36% (threshold positivity < 40%). Its twin was seronegative.

**Figure 6:**
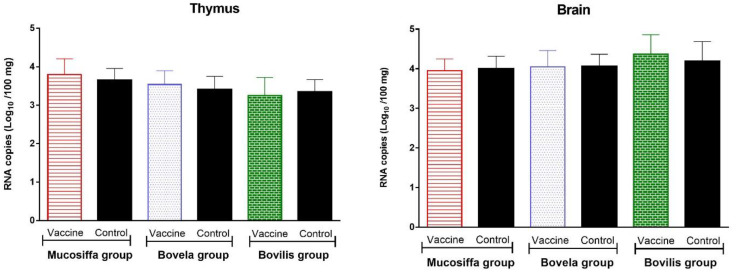
BDV-6 RNA loads (Log_10_/100 mg of tissue) in the thymus and the brain of fetuses sampled at D+66 of euthanasia from vaccinated (Mucosiffa in red, Bovela in blue, Bovilis in green) and unvaccinated (in dark) ewes.

RT-qPCR results showed that all seronegative fetuses were positive for BDV/BVDV in both brain and thymus samples, with Ct values ranging from 22.5 to 30.6 (Figure 6). The seropositive fetus was RT-qPCR negative in both sample types. The average viral loads in the brain were 3.9 ± 0.3, 4.1 ± 0.4, 4.4 ± 0.5 and 4.1 ± 0.4 Log_10_ RNA copies per 100 mg of tissue for the Mucosiffa, Bovela, Bovilis, and control groups, respectively. In the thymus, the loads were lower with 3.8 ± 0.4, 3.5 ± 0.4, 3.1 ± 0.6 and 3.3 ± 0.5 Log10 RNA copies per 100mg of tissue for the Mucosiffa, Bovela, Bovilis and control groups, respectively (Figure 6). There were no significant differences between groups (one-way ANOVA with Bonferroni correction).

Forty brain and thymus positive RNA samples were tested using another RT-qPCR kit (Taqvet BVD, Lsi, Life technology) to confirm the results. In addition, all tissue samples were sent to an independent laboratory (IdVet laboratory) for similar but independent RT-qPCR analyses, using two different BVDV RNA targets. All results fully validated the detection of BDV/BVDV in vaccinated and non-vaccinated fetuses. Confirmation of the challenge BDV-6 strain was done by sequencing Npro and 5′UTR of four RT-qPCR positive tissue samples (one of each group).

## 4. Discussion

The BDV-6 detection in the blood of unvaccinated ewes after infection and their seroconversion validate the model used in this study. The absence of disease in our infected ewes is classically described in experiments under standardized conditions [11,15,16]. This is consistent with local field observations, which showed that morbidity and mortality occur mainly after secondary respiratory and/or digestive infections. This is what has been described in the fattening workshops of young sheep in the Roquefort basin during the 2010–2013 epizootic [6]. These superinfections are explained by the capacity of BDV to induce a transient but severe leukopenia [11,16,17,18]. In this study, the BDV-6 challenge induced a marked leukopenia between D+2 and at least D+10 in naïve sheep. García-Pérez et al. [16] also observed that BDV-4 induced leukopenia but it was significant only between 2 and 5 days post-infection, suggesting that the duration and intensity of leukopenia may depend on the strain inoculated. Although BVDV vaccination does not quantitatively reduce leukopenia at the D+3 peak, it has the advantage of reducing its duration, since leukocyte levels return to normal values at D+6 for the two attenuated Mucosiffa and Bovela vaccines and at D+8 for Bovilis, whereas at D14 leukopenia was still observed in some sheep of the control group. Theoretically, since vaccination, especially with Bovela and Mucosiffa, reduces the period of immunosuppression, it could consequently reduce the window of susceptibility to concomitant infections. However, this partial protection is lower than the complete protection observed when cattle were vaccinated with the full dose of Mucosiffa and subsequently challenged with a BVDV-1 strain [19]. On the farm, the impact of BVDV vaccines on protection against leucopenia and secondary infections in sheep needs to be determined.

In cattle, control vaccination programs against BVDV involved using different types of live attenuated (MLV) and killed vaccines [20]. MLV vaccines have been shown to induce a full immune response, including neutralizing antibody response and T cell-mediated immune response, with solid fetal protection [20,21,22]. They also provide a longer duration of protection from clinical disease than inactivated vaccines [23]. However, they are considered less safe as vaccine strains may revert to virulence, recombine with wild-type strains and, for ncp strains, infect fetuses when injected in pregnant cows. Killed vaccines are viewed as being safer than MLV vaccines, they induce high antibody titers, but they are not as effective at inducing T cell-mediated immunity and providing protection from a diversity of BVDV strains [24,25,26]. Fetal protection after vaccination with killed vaccines varies from incomplete to satisfactory [27,28,29]. In this study, we tested two MLV and one killed vaccine commonly used for BVDV and BD control in France. The MLV Mucosiffa vaccine is considered safe since it contains a live-attenuated cp BVDV-1a strain (Oregon C 24 V) which is not able to infect the fetus and establish a persistent infection. More recently, the MLV Bovela vaccine has been developed based on genetic modification of two ncp BVDV-1 and BVDV-2 strains that both exhibited a combination of a deletion of a large part of the N(pro) gene and a deletion of codon 349 of the Erns gene, which abrogates the RNase activity of the structural glycoprotein [30]. Reports indicated that this vaccine-induced strong humoral and cell immune responses without infecting the fetus [30]. A recent work, however, reported the ability of the Bovela vaccine virus to cross the placenta inducing the presence of the vaccine virus RNA and antigen for an extended period only in the skin of the newborn calves and without viremia or excretion of the vaccine strains [31]. The Bovilis-BVD aluminum-adjuvanted killed vaccine used in this study contains the inactivated C-86 BVDV-1 cp strain. All three vaccines were previously shown to be efficacious for fetal protection against a BVDV-1 challenge in cattle [19,30], the Bovela vaccine was also successful for fetal protection against a BVDV-2 challenge [30].

Since vaccination of sheep with half doses of BVDV vaccines is a common practice for BD control in France, associated or not with PI lambs’ removal, we choose to immunize ewes according to the same protocol, assuming that at least for the two live vaccines this would have less impact on the immune response. The attenuated Mucosiffa or Bovela vaccine strains replicated in the host and therefore induced a serological ELISA response against NS3 in a large majority but not all vaccinated ewes. In addition, a small number of ewes, vaccinated or not, also appeared intermittently seronegative for NS3 after challenge, between D+35 and D+66. In contrast to attenuated vaccines, vaccination with Bovilis did not result in NS3 seroconversion at D0. The Bovilis vaccination however induced an anamnestic immune response, as NS3 seroconversion after challenge occurred significantly earlier in this group than that observed in unvaccinated ewes. Studies in cattle have also shown that some but not all inactivated BVDV vaccines do not induce detectable antibodies against the NS3 proteins [32,33] and they can be considered, at least under certain conditions [34], as DIVA vaccines (able to distinguish vaccinated and/or infected). However, other studies showed that the presence of non-structural proteins in the crude virus preparations used in inactivated vaccines caused subsequent NS3 seroconversion in vaccinated cattle [35,36]. In our study, the low number of animals and the fact that a small number of ewes, vaccinated or not, appeared intermittently seronegative for NS3 after challenge (between D+35 and D+66) suggests the need for further studies to investigate the NS3 ELISA response in a DIVA vaccine strategy.

In contrast to the NS3 ELISA response, all BVDV vaccines were able to induce a similar neutralizing antibody response against BDVD1a NADL strain after immunization of sheep, regardless of the genotypes 1a (Mucosiffa, Bovilis) or 1b (Bovela) of the vaccine strains. Induced detectable levels of cross-reacting antibodies against at least one other subgenotype have been previously described after BVDV-1 vaccination in cattle (for review, [37]) and BDV infection of sheep [9,11,16]. Our results however differ from those of Sozzi et al. who showed that the BVDV-1b vaccine strain of Bovela did not induce neutralizing antibodies against the BVDV-1a NADL strain after immunization of cattle [38]. We do not know if this difference is related to the SN method, to the ovine species, or to other factors. More surprisingly, cross-neutralization was also obtained after BVDV vaccination of ewes (D0) when BDV-6 strain was tested in SN, although VNT was lower than when using the BVDV1a NADL strain. This suggests that the heterologous SN response is lower than the homologous one. This is also confirmed by the VNT of the control ewes at D+66 that are higher than those of the vaccinated ewes at D0 when BDV-6 was used for SN.

The efficacy of BVD vaccines to protect sheep against BDV infection has never been experimentally investigated. Despite the presence of BDV-6 neutralizing antibodies at D0 in vaccinated ewes, BVDV vaccination was not sufficient to prevent fetal infection by BDV-6 in our challenge model as confirmed by the virus detection in all fetuses except for one. As the fetuses were negative for BVDV/BDV antibodies, this means that they were infected prior to the development of lymphoid tissues and functional immune responses, resulting in tolerant PI animals. Only one fetus from an ewe vaccinated with Bovela was negative for BDV and positive for antibodies, suggesting that it was immunocompetent at fifty-two days of life and made an immune response against the challenge strain. The fact that the twin of this fetus was RT-qPCR positive for BDV confirms that the challenge strain passed the fetal barrier of the mother. The reason why BDV infection differed between the twin fetuses remains to be elucidated.

Several hypotheses can be put forward to explain the absence of fetal protection of BVD vaccines against BDV-6 challenge. The possible lack of protection may be related to the genetic distance between the BVDV vaccine strains and the BDV-6 challenge strain. The genetic and antigenic diversity of pestiviruses is well known and well documented in the literature. A good cross-protection has been observed within the wide range of BVDV-1 genotypes, as was cross-protection against BVDV-2 using BVDV-1 based MLV vaccines [37,39]. However, the inability to fully prevent fetal infection, postnatal infection, and virus shedding has, in part at least, been attributed to antigenic diversity [40,41,42]. The genetic distance between BDV and BVDV is greater than that between the two BVDV genotypes, which may explain the failure of cross-protection after vaccination. This seems a weak hypothesis since all three vaccines induced the production of neutralizing antibodies against the two different viral species.

In this study, we did not investigate the CD4+ and CD8+ T-cell immune response, which was speculated in cattle to also drive the clinical cross-protection at least observed by *Pestivirus* A vaccines [37]. The cytotoxic CD8+ response eliminates BVDV infected cells while CD4+ T helper cells are key players in the development of protective humoral and cellular immunity against the virus. These cells mainly target the NS3 and E2 proteins but also other proteins such as the Npro, C, and Erns proteins [43,44,45,46]. As E2 glycoprotein is the most divergent between *Pestiviruses,* we could hypothesize a lack of T-cell cross-reaction for E2 between the BVDV-1 vaccine and the BDV-6 challenge strains. On the other hand, E2 is also the main target for neutralizing antibody response which was shown to cross-react in this study, albeit neutralizing and T-cell epitopes of E2 are different. In addition, the highly conserved NS3 is known to induce cross-reactivity for T-cells and will probably generate recall T cell responses at least in sheep vaccinated with the two MLV vaccines [37]. The role of the cellular response for the prevention of BDV fetal infection remains to be determined, especially whether BDV crosses the synepitheliochorial ovine placenta in the form of free virus and/or through the passage of infected cells.

The neutralizing antibody response against BVDV has been widely used as an important parameter to evaluate the immune responses for both vaccination and natural infection [20,37]. Neutralizing antibodies mainly target the E2 surface glycoprotein, and to a lesser extent to the Erns protein [20]. They were shown to bind free viruses where they play a major role in inhibiting the attachment of the virus onto a target/host cell [46]. In this study, the three used BVDV vaccines were able to induce a cross-neutralizing antibody response in sheep but this did not prevent the fetal infection. More information is necessary to explain how the antibodies are mediating their effect to prevent fetal infection. Some antibodies might just block binding but not affect their functional activity allowing for passage through the placenta. In this case, it should be determined why this happens when the three vaccines are injected in sheep and not when they are used in cattle [19,30]. Another explanation is that, as we use a severe challenge model with an IM injection of BDV-6 (2 × 10^5^ TCID_50_/ewe), this could contribute to the rapid release of large amounts of virus into the blood and thus exceed the neutralization capabilities of antibodies present at D0. In a previous study, we showed that cows vaccinated with Mucosiffa, which had VNT between 7.5 to 9.2 Log2 ED50/mL at the day of challenge (using the same SN assay), were fully protected for fetal infection against a severe BVDV-1 Han challenge by intranasal route [19]. In this experiment, VNT against BDV-6 at D0 ranged only between 4.7 to 6.5 Log2 ED50/mL, which could contribute to the failure of protection. The relationship between VNT and protection remains controversial [9,13]. Beer et al. [47] showed that a ≥ 1:512 VNT was required for marked protection against BVDV infection in cattle, although a VNT of 1:256 was found to be critical for the prevention of clinical signs [48]. It is, however, speculative to compare these thresholds with our data as the methods are different. In our SN assay, protection of all cell culture wells by a serum dilution between 1:256 and 1:512 would correspond to a VNT threshold of protection between 12.7 to 13.7 Log_2_ ED50/mL, which is not consistent with the total protection we previously obtained in cattle with VNT between 7.5 to 9.2 Log2 ED50/mL [19].

Finally, in field conditions, the BVDV or BDV loads in the blood at a given time depend on many factors such as the mode of contamination (sources of infections, contamination patterns, routes of infection, etc.) the host susceptibility, and the viral strain. If we assume a minimal threshold of neutralizing antibodies at a given time to protect against fetal infection, a severe infection, as described in our challenge model, may exceed the neutralizing response and may explain some sheep vaccination failures we observed in the field with BVDV vaccines. On the other hand, in some situations, vaccination was reported by farmers as effective in the field to control BD, with or without associated measures of PI removal (personal communications). It would be interesting to confirm these assertions by evaluating whether a heterologous degree of protection exists in the case of infection of vaccinated sheep by low viral loads. In any case, BVDV vaccination could only be considered as a complementary measure in the control of BD. This also raises the interest in having future vaccines with BDV valences.

## Figures and Tables

**Table 1:** Percentages of vaccinated (Mucosiffa, Bovela, Bovilis) and unvaccinated ewes which are seropositive for BDV NS3 antibodies (BVD p80 (NS2-3) antibody One-Step kit, IDvet, Montpellier, France).

Groups/Days	D0	D+7	D+14	D+21	D+28	D+35	D+42	D+66
Mucosiffa (n = 11)	73%	73%	91%	91%	91%	91%	100%	100%
Bovela (n = 11)	64%	64%	100%	91%	82%	100%	91%	100%
Bovilis (n = 11)	0%	18%	64%	73%	82%	91%	82%	100%
Non vaccinated (n = 18)	0%	0%	0%	17%	67%	72%	94%	94%

## Data Availability

The data are available under reasonable request to the corresponding author.
